# Grizzly bear population genomics across a coastal–interior ecotone in British Columbia, Canada

**DOI:** 10.1093/g3journal/jkaf237

**Published:** 2025-10-07

**Authors:** Lauren H Henson, Kris A Christensen, Ben J G Sutherland, Hollie A Johnson, Bridgett vonHoldt, Astrid Vik Stronen, Paul C Paquet, Jason Moody, Ben F Koop, Chris T Darimont

**Affiliations:** Department of Geography, University of Victoria, Victoria, BC V8P 5C2, Canada; Raincoast Conservation Foundation, Sidney, BC V8L 2P6, Canada; Department of Biology, University of Victoria, Victoria, BC V8W 2Y2, Canada; Sutherland Bioinformatics, Lantzville, BC V0R 2H0, Canada; Department of Biology, University of Victoria, Victoria, BC V8W 2Y2, Canada; Department of Ecology and Evolutionary Biology, Princeton University, Princeton, NJ 08544, United States; Biotechnical Faculty, University of Ljubljana, Jamnikarjeva ulica 101, Ljubljana 1000, Slovenia; Department of Geography, University of Victoria, Victoria, BC V8P 5C2, Canada; Raincoast Conservation Foundation, Sidney, BC V8L 2P6, Canada; Nuxalk Stewardship Office, Bella Coola, BC V0T 1C0, Canada; Department of Biology, University of Victoria, Victoria, BC V8W 2Y2, Canada; Department of Geography, University of Victoria, Victoria, BC V8P 5C2, Canada; Raincoast Conservation Foundation, Sidney, BC V8L 2P6, Canada

**Keywords:** conservation genomics, grizzly bear, local adaptation, population genomics, single nucleotide polymorphism, whole genome resequencing

## Abstract

Local adaptation research often focuses on discrete populations without extensive gene flow that are under differential selective pressures. By contrast, grizzly bears *Ursus arctos* in British Columbia (BC) are wide-ranging omnivores that span an environmental and resource ecotone from the coastal, salmon-enriched rainforest to dry interior plateau. This ecotone has been associated with local adaptation in other species and the different regions to morphological variation in grizzly bears. To understand genome-wide population genetic patterns across the ecotone and to identify loci or genomic regions associated with these different environments, here we use whole-genome resequencing to characterize 3.9 M SNPs in 31 grizzly bears spanning from central to northern latitudes in coastal and interior regions (to the west and east of the coastal mountain range [CMR], respectively). Clustering grizzly samples by genotypes identified 3 groups that generally correspond to the source geographic regions, with the greatest variation occurring from north to south. The data were best explained by a single ancestry cluster, but *K* = 3 recovered the 3 geographic groupings and were used to identify putative nonmigrant individuals. The presence of individuals with mixed ancestry (using *K* = 3) provides evidence for travel across the CMR, but significant differentiation between clusters (mean *F*_ST_ = 0.015 to 0.036) suggests some genetic separation between the regions, supporting an isolation-by-distance or clinal variation model. Putative close-kin were identified and removed, then multiple supervised outlier SNP detection methods were applied to identify regions of the genome consistently segregating between coastal and interior regions. Several associated genomic regions and candidate genes were identified, including a consistently identified outlier region near the gene *creatine kinase, m-type*. This work provides the first genome-wide analysis of grizzly bears in the studied region. These findings will be useful for connectivity planning and research on the adaptability of coastal and interior grizzlies to future climate change scenarios.

## Teaser

The authors investigate grizzly bear population genomics between two different environments of British Columbia: the resource rich coastal rainforest and the dry, interior plateau. Using millions of genetic variants identified across the genome they group the sampled grizzlies into subpopulations that roughly correspond to their source sampling locations and use these groupings to compare the coastal and interior grizzlies across the Coast Mountain Range. Several genomic regions were associated with these different environments, which may provide insight into the differences between the subpopulations, and guide future work considering adaptability to climate change scenarios.

## Introduction

Local adaptation can occur due to evolutionary processes that provide fitness advantages to different populations in response to local environmental pressures (Kawecki and Ebert 2004; Blanquart et al. 2013), for example as a result of elevation (Halbritter et al. 2018) or salinity gradients (Sanford and Kelly 2011). This can be impacted by gene flow, as demonstrated in host-parasite interactions, where the relative rate of gene flow in host and parasite can determine local adaptation outcomes (Hoeksema and Forde 2008). Although local adaptation is typically documented in case studies with limited or no gene flow, it can also occur at microgeographic scales and with gene flow among populations (Richardson et al. 2014; Tigano and Friesen 2016). In some cases, specific regions of the genome can be associated with significant phenotypic differences even though the rest of the genome is undifferentiated (e.g. run timing; Barry et al. 2024), and this highlights the importance of considering genome-wide data when investigating local adaptation.

In the presence of gene flow, ecotones (i.e. transition areas between ecological communities) can foster local adaptation (Wright et al. 2006; Yuan et al. 2018; Ekar et al. 2019). For example, substrate coloration differences along the ecotone of White Sands, New Mexico (United States), where the white gypsum geological feature transitions from lighter to darker soil, resulted in dorsal color variation within populations of 3 local lizard species (Rosenblum 2006). In reciprocal transplantation experiments of eastern oyster (*Crassostrea virginica*) along the lagoon ecotone in eastern Florida (United States), reciprocal home-site advantage occurs and signatures of local adaptation are present (Burford et al. 2014). Ecotone transition zones can have pronounced environmental gradients, and this can exert different selective pressures depending on the location across the gradient in which the population resides.

Detecting local adaptation has become increasingly possible with rapid advances in genomics (Hoban et al. 2016). Although local adaptation was previously investigated by reciprocal transplants (an approach not possible for all species), genomic datasets now allow the detection of genomic patterns underlying adaptive diversity (Funk et al. 2019). Loci that are putatively adaptive can be identified through association tests of allele frequencies and environmental variation. When environmental drivers of local adaptation are unknown, genome scans can contrast across populations to identify candidate outlier loci with elevated differentiation (Hoban et al. 2016). This is possible with relatively few samples, but understanding the role of the associated loci often requires an investigation in a larger proportion of the population (Lotterhos and Whitlock 2015). Genome scans have identified signatures of local adaptation in response to habitat differentiation, latitudinal gradients, and environmental clines in many species, including the Eurasian blue tit *Cyanistes caeruleus* (Perrier et al. 2020), the Mediterranean striped mullet *Mullus surmuletus* (Dalongeville et al. 2018), and the thick-billed murre *Uria lomvia* (Tigano et al. 2017).

A dramatic ecotone in British Columbia (BC), Canada, spans the coastal rainforest environment through the dry interior plateau and provides the necessary conditions to foster intraspecific variation. These disparate environments are divided by the coast mountain range (CMR). The CMR hosts a hybrid zone for subspecies of the Swainson's thrush *Catharus ustulatus* (Ruegg 2008), a region of intraspecific migratory, morphological, and genetic variation in the Hermit thrush *Catharus guttatus* (Alvarado et al. 2014), and an area of introgression between white spruce *Picea glauca* and Sitka spruce *Picea sitchensis* (Bennuah et al. 2004). Even highly mobile mammals show divergence across this ecotone; the grey wolves *Canis lupus* of coastal BC are highly differentiated from interior populations and represent a unique ecotype and evolutionarily significant unit (Muñoz-Fuentes et al. 2009; Schweizer et al. 2016b) with genetic differences in genomic regions related to dietary fat and lipid metabolism and coat color (Schweizer et al. 2016a). Other differences exist in grey wolves across this ecotone, including divergent mitochondrial genetics and differences in morphology and diet (Muñoz-Fuentes et al. 2009), where the coastal wolves forage extensively on salmon and other marine resources (Darimont et al. 2003). A major segregating environmental resource for the coastal–interior ecotone is from the various dietary resources available to generalist species (e.g. abundance and availability of salmonids *Salmonidae* spp.).

Salmonids are a defining resource for many species, ecosystems, and people in the coastal region of BC. Grizzly bears *Ursus arctos* are particularly reliant on salmon. Individual bears with increased access to salmon tend to be larger, have bigger litters, have lower stress hormone levels, and exist at higher densities at a population level (Rausch 1963; Hilderbrand et al. 1999; Bryan et al. 2013). Salmon also migrate into the interior (Quinn 2018), but contemporary salmon availability and consumption decline significantly past the CMR (Hilderbrand et al. 1996; Adams et al. 2017, 2024). Phenotypic differences occur across the coastal–interior ecotone in grizzlies, with interior grizzlies having smaller body mass and skull size than coastal individuals (Rausch 1963; Kurtén 1973; Paetkau et al. 1998). The potential causes of this phenotypic difference are not fully understood. It may be related to phenotypic plasticity and salmon consumption (Hilderbrand et al. 1999); larger males require high-protein food to gain mass (Robbins et al. 2007), and smaller bears can gain mass through vegetation consumption alone (Felicetti et al. 2003). Alternatively, since interior bears have long existed without widely available aggregations of high-quality food like salmon, they may have adapted to intermittent availability of meat resources, contrasting the coastal grizzlies that depend on salmon to support their larger body mass (Mowat and Heard 2006). Size differences between some coastal and interior populations could also be influenced by differential introgression of polar bear *Ursus maritimus* alleles through past hybridization events (Cahill et al. 2015, 2018; Miller et al. 2024), although a potential role for this in coastal BC has not been described. The clinal variation in resources and associated morphology in grizzly bears across the BC coastal-to-interior ecotone makes it a valuable system to investigate genomic signatures of local adaptation in this wide-ranging species.

Population genomics and local adaptation can be used to inform management and policy (Waples et al. 2022) and are expected to support the assessment of the provincially managed Grizzly Bear Population Units (GBPUs; Province of British Columbia 2012), and management activities of Indigenous Stewardship Offices. Local Indigenous ecological knowledge indicates that interior bears migrate from the interior to the coastal areas through the Bella Coola Valley, Nuxalk Territory (e.g. Stuie; 52.3699°N, 126.0659°W) to access resources, including salmon (Jason Moody, Nuxalk Nation, *pers. comm*). Therefore, we hypothesize that the Bella Coola Valley and other valleys like it that transect the CMR may provide opportunities for gene flow across the ecotone. Furthermore, the ecotone may provide environmental resource gradients that could foster local adaptation at the extremes of each region, and so here we aim to identify genomic loci or regions of elevated segregating genetic variation between the coastal and interior regions. Collectively, this work provides new genomic resources and insights regarding the population genetics of grizzly bears in BC, and on the presence of and genomics underlying putative local adaptation across the coastal-to-interior ecotone. This work also provides insights for future work regarding the relevance of adaptive variation in grizzly bear conservation and resilience to future climate scenarios.

## Methods

### Sample collection, DNA extraction, library preparation, and sequencing

**Fig. 1.**
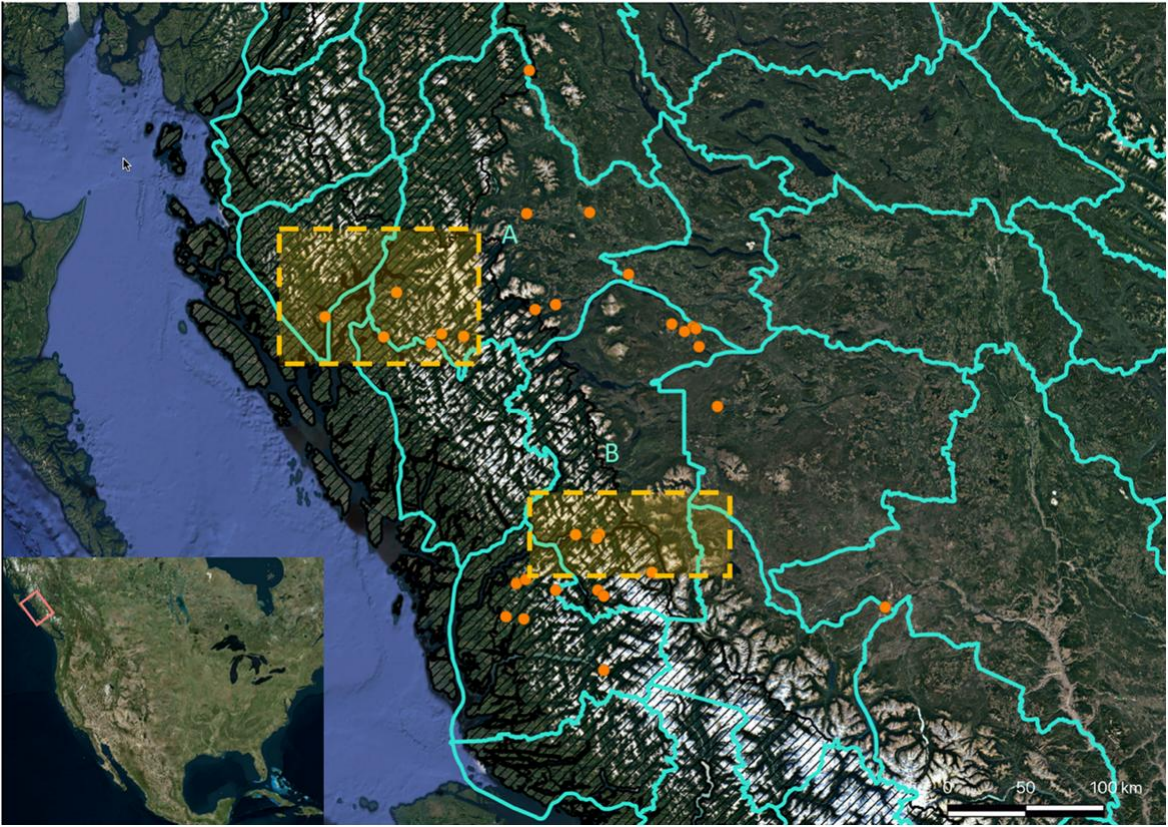
The study area including the Central Coast and adjacent interior of British Columbia (BC). The coastal ecoregions are shown with a black hash, and grizzly bear sampling locations are indicated by orange points (*n* = 31, samples from 1996 to 2016). GBPU boundaries are shown in teal, and the Bulkley and Tweedsmuir GBPUs are indicated with a or b, respectively. The yellow dashed boxes indicate the approximate areas of the Bella Coola Valley (South) and valleys containing Kitimat and Kemano (North). The inset situates the study area within North America. This map was generated in ArcGIS.

Dried hide samples were obtained from the BC Ministry of Environment compulsory inspection database of killed grizzly bears through a data share agreement. Samples were selected from the Central Coast and adjacent interior region to represent the coastal-to-interior plateau ecotone of BC (Fig. 1). Samples were collected from 1996 to 2016. Geographic coordinates provided per sample are slightly offset (jittered) locations of where each bear was killed (Supplementary Additional File 1), as per requirements of the data share agreement. Locations were plotted using ArcGIS (Esri Inc.). The recorded phenotypic sex of the selected samples (*n* = 31) included 9 females and 22 males, a sex ratio that reflects the higher frequency of males in killed bears.

Genomic DNA was extracted from hide tissue using the DNeasy Blood and Tissue kit (QIAGEN) from 25 mg slivers of dried tissue following the manufacturer's guidelines, but with an overnight incubation in lysis buffer and a second separate elution from the columns. Purified genomic DNA was quantified using spectrophotometry (Nanodrop; ThermoFisher) and fluorimetry by Qubit dsDNA-BR (ThermoFisher). Samples were submitted to the Génome Québec Innovation Centre for PCR-free shotgun whole-genome library preparation to be sequenced on HiSeqX and NovaSeq6000 S4 (Illumina) using paired-end 150 bp reads to a per-sample target depth of 10× coverage.

### Variant calling and filtering

Variants were called using the GATK pipeline (Van der Auwera et al. 2013) as described below and in the associated code repository (see Data availability). Paired-end reads were aligned to the *Ursus arctos* reference genome (GCF_003584765.1; Taylor et al. 2018) using *bwa mem* (v.0.7.17; Li 2013). Read groups were added with experimental information using the Picard Toolkit function *AddOrReplaceReadGroups* (v.2.18/9; Broad Institute 2024). Alignments were sorted and indexed using SAMtools (v.1.9; Danecek et al. 2021). Alignment rates were calculated based on the number of alignments passing a minimum threshold of at least 100 bp alignment with a minimum percent identity of 98%, expressed as a fraction of the total aligned reads (see Data availability). PCR duplicates were identified and marked using the Picard Toolkit function *MarkDuplicates*, and samples that were split between sequencing lanes were merged based on read group identifiers.

All GATK batch scripts are provided (see Data availability) and described here in brief. Haplotypes were called with the GATK *HaplotypeCaller* using flags *genotyping_mode* DISCOVERY and *emitRefConfidence* GVCF. Genotypes were extracted from the resulting GVCF files using the function *GenotypeGVCF* at intervals of 10 Mbp. The resultant files were merged using the function *CatVariants*. All merged files were sorted using the *vcf-sort* function of VCFtools (Danecek et al. 2011). Variants were then scored using the *VariantRecalibrator* in SNP mode, and *ApplyRecalibration* functions of GATK. Filtering of variants was conducted using VCFtools to remove indels and to only retain biallelic SNP variants with a minor allele frequency (MAF) ≥ 0.05. SNPs were filtered to keep those with less than 10% missing data across samples.

After SNP calling was completed and during project analysis, a new chromosome-level reference genome became available (GCF_023065955.2; UrsArc2.0; Armstrong et al. 2022). To make use of the updated genome, with improved metrics, annotation, and assembly of sex chromosomes, the program SNPlift (Normandeau et al. 2023) was used to transfer SNP positions from the reference genome used for calling SNPs to the new assembly (UrsArc2.0). The output VCF file was provided a new header with the bcftools function *reheader*. Variants oriented to the UrsArc2.0 assembly were used for all downstream applications.

SNPs transferred to UrsArc2.0 then underwent additional filtering to remove SNPs within 5 bp of indels, only keeping SNPs with an overall quality score (i.e. QUAL) of at least 20 and an average read depth across all samples ≥7. Subsequently, all genotypes per individual supported by <5 reads or >1,000 reads were set to missing values, as were any genotypes with individual quality scores (GQ) < 20; SNPs were then filtered again to only retain SNPs missing in <15% of individuals. A final all SNPs dataset was generated by reapplying the MAF filter (MAF > 0.05), and an LD-filtered dataset was generated for population genetic purposes by removing SNPs based on linkage (i.e. keep if linkage < 0.5 in 50 kbp windows) using bcftools (Danecek et al. 2021).

### Mitochondrial DNA haplotypes and phylogenetics

To fit the samples from the current study into a broader phylogenetic context with previous work, the multiple alignment program MAFFT (v.7; Katoh et al. 2019) was used to align a 701 bp fragment of the mitochondrial control region for each of the 4 unique haplotypes from the 31 samples, 5 haplotypes from coastal Alaska (Talbot et al. 2014), and all 80 previously published haplotype sequences representing grizzly bear mitochondrial clades 1 to 6 from Miller et al. (2006) to the reference genome (Taylor et al. 2018). A phylogenetic tree was generated using the MEGAX program (Kumar et al. 2018) with the Maximum Likelihood method and the Tamura-Nei model (Tamura and Nei 1993) applying Neighbor-Join and BioNJ algorithms. An American black bear *Ursus americanus* control region sequence was used as the outgroup (Miller et al. 2006).

### Genetic characterization, relatedness, and genetic sex

The LD-filtered VCF file was used for population genetic characterization (see Data availability). To avoid impacts of sex-linked loci (Benestan et al. 2017), SNPs on the sex chromosomes were removed using bcftools to create an autosome-only dataset (any SNPs on the mitochondrial genome were also removed). The VCF file was loaded into the R environment (R Core Team 2025) and converted to genind format using vcfR (Knaus and Grünwald 2017) to be analyzed using the *simple_pop_stats* repository (see Data availability). The genind file was converted to genlight format to conduct a principal component analysis (PCA) through the gi2gl function of dartR (Gruber et al. 2018), followed by the glPca function of adegenet (Jombart and Ahmed 2011). PCA results were plotted using ggplot2 (Wickham 2016), with various sample metadata overlayed to inspect general trends, including percentage of missing data, genetic sex, geographic location of sampling, and year of sampling.

The dataset was converted to demerelate format with dartR, then converted to related format (Pew et al. 2015) using the related function *readgenotypedata*. The relatedness between individuals was calculated using the coancestry function of related, and the Ritland relatedness statistic (Ritland 1996) was used to interpret inter-individual relatedness. Outlier relatedness levels were determined by using *boxplot.stats* of R, and a relatedness cutoff value of 0.15 was used to consider pairs as having elevated relatedness. A single individual per pair with elevated relatedness was removed from the dataset until no pairs above the cutoff remained. A PCA was generated as described above using the close kin-removed dataset.

The genetic sex of individuals was confirmed against phenotypic (recorded) sex by taking raw reads for all 31 samples and aligning against the UrsArc2.0 assembly (GCF_023065955.2) using *bwa mem*, then sorting and indexing with SAMtools. The lengths of all chromosomes were calculated using custom Python code (see Data availability; E. Normandeau, Scripts), and the coverage across each chromosome was calculated using the genomeCoverageBed function of bedtools (Quinlan and Hall 2010). The average depths of coverage for the X and Y chromosomes were calculated, and the ratio of the coverage was determined to identify XX or XY individuals.

### Population substructure and cluster membership

The optimal number of clusters in the data was determined using several approaches. First, an unsupervised discriminant analysis of principal components (DAPC) was conducted, and the Bayesian information criterion (BIC) was observed for different numbers of clusters identified. A BIC elbow was sought, and scatterplots and assignplots were generated for 2 or 3 clusters, varying the number of retained PCs and discriminant functions and looking for stability in cluster membership. Second, ADMIXTURE (Alexander et al. 2009) was used from *K* = 1 to 6 (inclusive), with 10 replicate runs per value of *K* to obtain a per-*K* coefficient of variation (CV error) to identify the lowest CV error. StructureSelector (Li and Liu 2018) was used on these data to further identify the optimal number of clusters, using the ADMIXTURE output and either the estimated population identifiers from groupings on the PCA, or without different population identifiers.

The PCA, unsupervised DAPC, and ADMIXTURE results were collectively used to define the optimal number of clusters to explain the data. When individuals were assigned to similar clusters concordantly by multiple methods, they were considered assigned to the cluster. If discordant, the result for the individual was considered uncertain, and if the ADMIXTURE ancestry fraction was less than 70% for the major fraction, then the individual was also considered to have uncertain cluster membership and to have putatively mixed ancestry. Ancestry fractions and whether the individual was included in downstream analyses were plotted using QGIS (QGIS.org 2025). A dataset was then generated with only the individuals showing strong cluster membership to the 3 identified clusters. VCFtools was used to estimate the average *F*_ST_ between each cluster.

### Outlier detection across the coastal-to-interior ecotone

Outlier loci were investigated using the dataset with only the individuals having strong group membership (see above) but including all SNP loci (i.e. autosome-only, MAF > 0.05, no LD filter). As the focus of the study was the coastal-to-interior ecotone, the 3 identified clusters were defined as either coastal or interior for designating contrasts. First, a supervised DAPC was conducted, and loading values per locus were obtained; loci within the 99.99th percentile of loading values were considered to be associated with the regional differences. Loading values were plotted in a Manhattan plot in R using fastman (Paria et al. 2022), including only the scaffolds with at least 100 SNPs present in the full dataset to remove smaller scaffolds. Second, GEMMA (Zhou and Stephens 2012) was used with interior or coastal designations as binary values. A kinship matrix was generated among all individuals, and linear models based on the binary region definition per locus were conducted. Log-ratio test *P*-values were plotted in a Manhattan plot using fastman as described above, with a significance threshold determined by Bonferroni correction (i.e. 0.05 divided by the number of tests). Third, *pcadapt* (Luu et al. 2017) was used, and a screeplot and scoreplots for the first 6 principal components (PCs) were used to determine whether specific PCs separated the predefined clusters. The PC axis that best separated the coastal and interior groupings was determined and used as the relevant PC. Significance values (*P*-values) of loci associated with relevant PC produced by pcadapt were extracted, and a multiple test correction was applied using the p.adjust function in R using the Benjamini–Hochberg correction method to determine the significance of individual loci, then adjusted *P*-values were plotted in a Manhattan plot.

Comparisons of the different outlier SNP detection methods were conducted by identifying outlier loci and regions detected by multiple methods. Proximity to predicted genes for top outlier candidates was inspected using the annotation table from NCBI for the UrsArc2.0 reference genome. Genotypes of top outlier candidates were plotted based on allelic dosage in R.

## Results

### Whole-genome resequencing, genetic sex, and genotyping

The 31 unique grizzly bear samples had an average (± s.d.) number of reads per sample of 121.5 ± 43.5 M (Supplementary Additional File 1), or 15.9 ± 5.7× coverage, assuming a genome size of 2.3 Gbp. Alignment rates against the contig-level genome assembly (GCF_003584765.1) were on average 82.6 ± 4.9% of total reads. Following GATK genotyping (see *Methods*), 13,867,192 biallelic SNPs were identified. Applying an MAF filter resulted in the retention of 10,044,612 SNPs. Of these, 9,788,257 SNPs were transferred to the UrsArc2.0 genome assembly (see *Methods*). Removal of SNPs that were near indels or that had overall low quality or depth resulted in a minimal number of SNPs removed, and 9,683,999 SNPs were retained. Removal of low or very high depth or low-quality genotypes per individual (see *Methods*) resulted in a significant reduction of SNPs, suggesting the removal of many low-quality genotype calls (*n* = 3,895,954 SNPs retained). Following these filters, the samples had 9.6 ± 11.0% missing data overall (see Supplementary Fig. 1). The reapplication of an MAF filter resulted in 3,880,487 SNPs being retained in the all SNP dataset, and 327,820 SNPs in the LD-filtered dataset. SNPs on the sex chromosomes or mitochondrial genome were removed from the all SNP and LD-filtered datasets, resulting in the retention of 3,871,837 and 325,946 SNPs, respectively.

Raw read alignments to the UrsArc2.0 assembly were used to determine the genetic sex of individuals by analyzing alignments to the X (121.2 Mbp) and Y (30.9 Mbp) chromosomes (see *Methods*). Clear alignment differences were observed between the sexes; suspected females had very low relative average coverage on the Y chromosome (coverage of Y/X = 0.04), whereas suspected males had more similar coverage across both chromosomes (coverage of Y/X = 0.26). The determined genetic sexes matched phenotypic sexes provided with the samples, which specified 9 females and 22 males.

### Mitochondrial phylogeny of samples within known clades

Following the definition of clades identified in Miller et al. (2006), our analysis of previously analyzed haplotypes (Miller et al. 2006) with samples from coastal Alaska (Talbot et al. 2014) and haplotypes identified in our samples resulted in a tree where all clades except for Clade 6 were largely retained (Supplementary Fig. 2). In our results, Clade 6 was split into 2 paraphyletic clades, with 2 samples together with Clade 2 and 3 samples within their own clade. The mitochondrial haplotypes identified in the target mitochondrial region from the samples of the present study (*n* = 4 unique haplotypes) formed a smaller clade with haplotypes from eastern Russia, coastal Alaska, and Central Coast BC (Supplementary Fig. 2). This smaller clade clustered within the larger clade with other haplotypes from Alaska and BC, broadly within Clade 3 (Miller et al. 2006; Talbot et al. 2014). Clade 3 has previously been separated into Clade 3a (Eurasia, Alaska, and Hokkaido Central), 3b (Canada and Hokkaido East), and 3c (Middle East) (de Jong et al. 2023). Clade 3b, as defined in Miller et al. (2006), contains haplotypes 66, 67, and R252 (see Fig. 1 of Miller et al. 2006), and our samples clustered with these Clade 3b haplotypes (Supplementary Fig. 2).

### Population and sample characterization

**Fig. 2.**
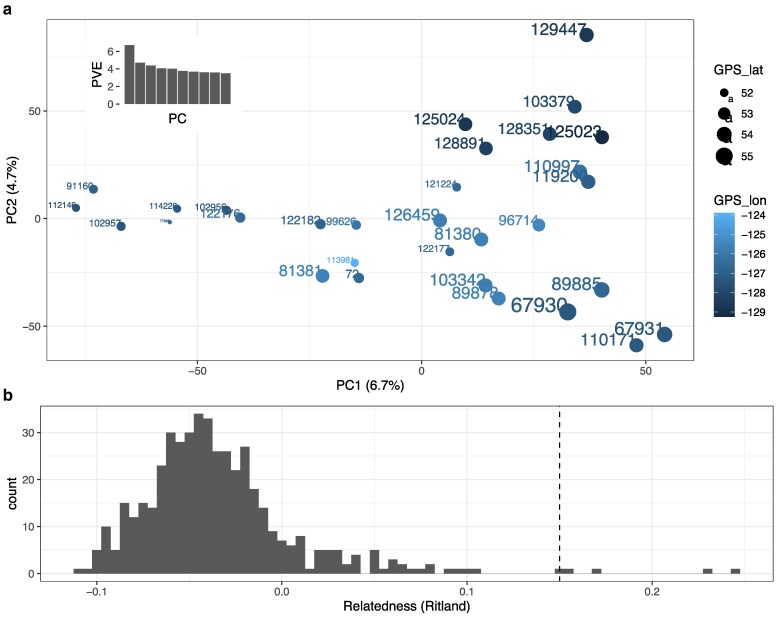
a) Samples clustered by PCA based on genotypes including all individuals (*n* = 31) and linkage-filtered SNPs. Sampling site geographic locations are indicated by point size (latitude) and color (longitude). b) Relatedness distribution of pairs of all grizzly bears in the analysis. The hatched vertical line indicates Ritland metric of 0.15, the cutoff applied defining pairs with elevated relatedness.

Using PCA on the LD-filtered genotypes resulted in individuals generally clustering by the geographic location of sampling (Fig. 2a). PC1 separated individuals across latitude (southern = negative PC1; northern = positive PC1; percent variance explained, PVE = 6.7%). PC2 separated across longitude, with more western (i.e. coastal) samples in positive PC2 and eastern (interior) samples in negative PC2 (PVE = 4.7%). These clusters included individuals of both sexes and from a wide variety of sampled years (Supplementary Fig. 3), suggesting temporal stability. Some notable exceptions to these trends were observed. Two samples from the southern end of the sampled area (i.e. 121224 and 122177 from south of Bella Coola) clustered closer to the more northern samples. A sample collected from the furthest eastern location in the dataset and one of the furthest south sampling points (i.e. 113981 from the Chilcotin Region) was positioned in the middle of the PCA sample distribution. However, generally, the overall groupings corresponded to geographic location of sample collections. Inspecting sex, year of collection, or percentage of missing data did not explain PCA clustering (Supplementary Fig. 3).

Genetic relatedness between samples was estimated and paired relatedness values were found to generally follow a normal distribution with a right tail representing pairs with elevated relatedness (Fig. 2b). Four pairs involving 5 unique individuals exhibited relatedness estimates above the set cutoff (Ritland metric > 0.15; Supplementary Table 1). Notably, these pairs were comprised of individuals sampled geographically close to each other. The most closely related pair (i.e. 110171 and 67931; Ritland = 0.2448) was comprised of two males sampled from the western arm of the Nechako reservoir in 2010 and north of Morice River in 1996, respectively (distance between = 61.5 km; Supplementary Table 1). The other highly related pairs were sampled near Rivers Inlet. Individuals 112146 (female, 2010) and 114228 (male, 2014; Ritland = 0.2149) were sampled in the mountains north of Rivers Inlet approximately 12 km apart, and 102956 (male, 2009), also estimated to be related to this pair (e.g. 114228 to 102956 Ritland = 0.1816), was sampled approximately 23 km to the north. As expected, the individuals with elevated relatedness also clustered closely in the PCA (Fig. 2a). Individuals 102956 and 114228 were among those samples with higher levels of missing data, but all the others with high relatedness did not have higher levels of missing data (Supplementary Table 1; Supplementary Additional File 1), suggesting that the relatedness trend was not caused by missing or low-quality genotypes. Three samples were removed from the dataset to avoid impacts of putative close-kin (i.e. 110171, 114228, 102956), but this did not significantly impact clustering.

### Defining population clusters

Population structure was investigated using an unsupervised DAPC and ADMIXTURE (*n* = 28 individuals; 325,946 SNPs). The DAPC identified a slight elbow in the BIC at *K* = 2 (Supplementary Fig. 4). Furthermore, DAPC assignment of individuals to clusters was more stable at *K* = 2 than *K* = 3; when using *K* = 3, slight variations in the number of retained PCs in the DAPC resulted in large differences in cluster formation, where the third cluster was frequently comprised of only a few individuals. Therefore, *K* = 2 was used to assign individuals to clusters by DAPC (Supplementary Fig. 5).

**Fig. 3.**
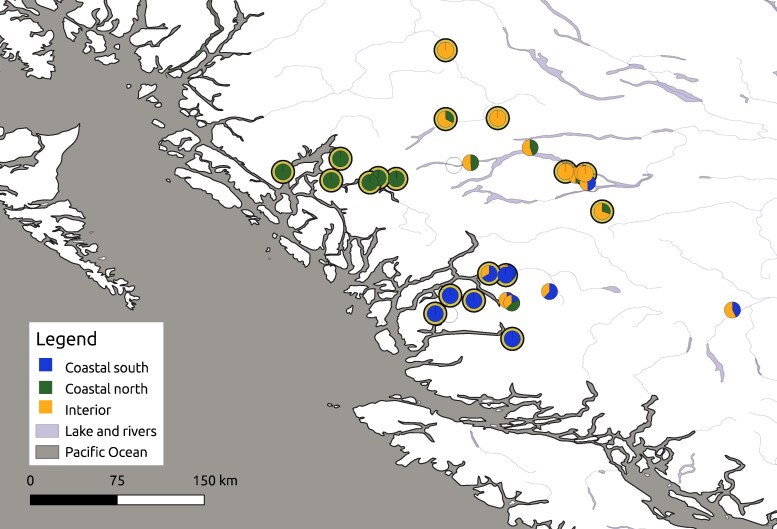
ADMIXTURE fractions plotted per sample along with GPS coordinates. Pie charts are shown with their ADMIXTURE fractions, and those with a yellow ring outline were retained for outlier loci detection analysis. Open circles are samples that were removed due to high relatedness (*n* = 3). This map was generated with QGIS.

The lowest ADMIXTURE CV error occurred with *K* = 1 (Supplementary Fig. 6), suggesting that all samples descend from one main ancestry cluster. However, to investigate potential substructure related to the groupings observed in the PCA (see above), and to identify individuals with strong cluster membership to the 3 PCA groupings observed, we explored *K* = 3. This analysis separated samples largely into the 3 groupings observed in the PCA (coastal south, coastal north, and interior; Fig. 3; Supplementary Fig. 7b).

Using ADMIXTURE *K* = 3 and considering only individuals with ancestry fractions greater than 70% as well as consistent groupings by both ADMIXTURE and DAPC, the coastal south, coastal north, and interior clusters contained 6 individuals each (*n* = 18 total; Fig. 3; Supplementary Fig. 7b). Individuals 122177 and 72 showed discrepancies between the methods in cluster assignment, and so even though they had >70% ancestry fractions, they were not retained in the interior grouping.

Although sample sizes were generally low, to understand the extent of genetic differentiation between these groupings, average *F*_ST_ was evaluated between groups. The differentiation analysis was in concordance with the PCA in that the greatest difference was observed by latitude, with north coastal and south coastal *F*_ST_ = 0.036, relative to north coastal and interior being *F*_ST_ = 0.015 (Supplementary Table 2).

### Outlier detection and genomic characterization

**Fig. 4.**
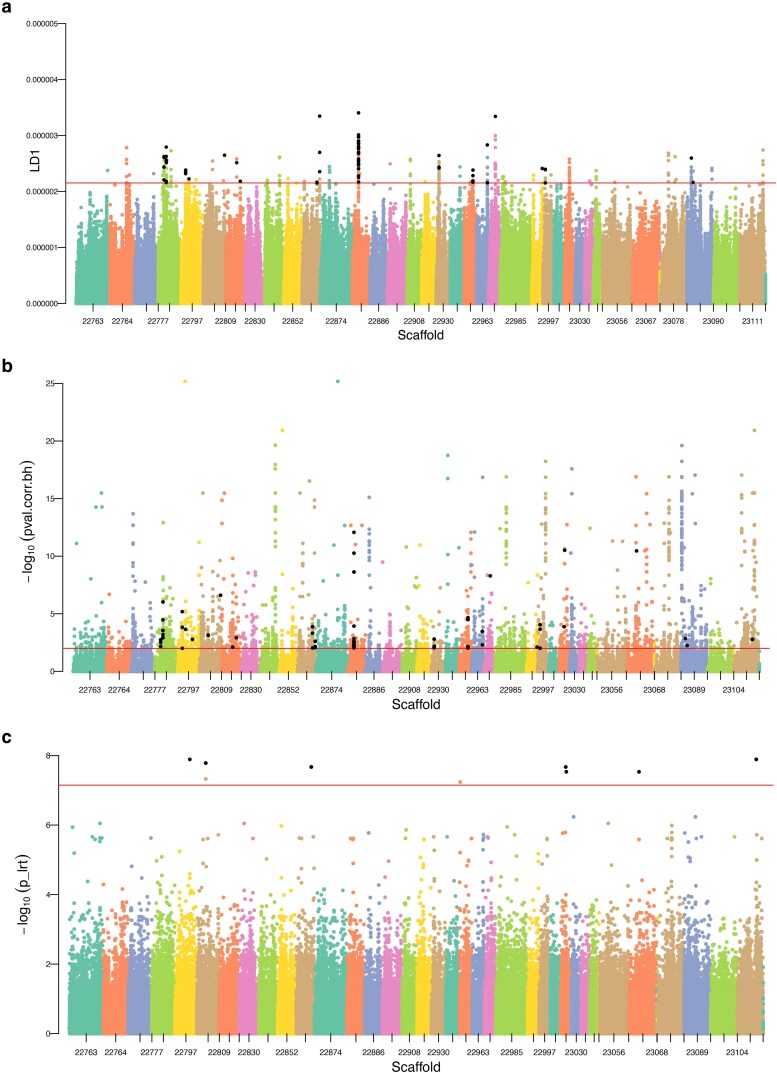
Manhattan plots for coastal vs inland comparisons showing putative outlier SNPs using a) DAPC, where the horizontal line indicates top 99.99th percentile; b) pcadapt, where significance is Benjamini–Hochberg corrected *P* < 0.01 based on PC2 only; and c) GEMMA, where significance is Bonferroni MTC *P* ≤ 0.05. Scaffolds with at least 100 SNPs present in full dataset are shown, with prefix (NW_0266) removed. Black dots are highlighted SNPs that have been identified by multiple methods.

Outlier identification was conducted through supervised analyses of coastal (coastal north and coastal south; *n* = 12) and interior (*n* = 6) groupings using several methods. The supervised DAPC approach used 3,858,384 loci and resulted in discrete separation between the groups across discriminant function (DF) 1 (Supplementary Fig. 8). The 0.01% of loci with top loading values were identified (*n* = 386 SNPs), and these had loadings per allele ranging from 2.15E−6 to 3.40E−6 (median loading contribution of all loci = 5.4E−8; median of top 99.99th percentile loci = 2.4E−6; i.e. 44.0× higher median in the outliers). These top outliers were found throughout the genome, including regions with multiple SNPs observed in peaks (Fig. 4a). The pcadapt approach used 3,706,201 loci and found PC1 to explain the latitudinal separation, as was observed for PC1 in the main PCA (see above), and PC2 to explain the coastal/interior separation (Supplementary Fig. 9). Significant outlier SNPs (Benjamini–Hochberg corrected *P* < 0.01) were identified that were associated with PC2 (*n* = 4,391 SNPs). These loci were also found throughout the genome, with some having very low *P*-values (Fig. 4b). The GEMMA approach analyzed 703,001 loci and found 24 SNPs to be significantly associated with the coastal/interior separation (Bonferroni-adjusted *P* ≤ 0.05). These were found on 7 different scaffolds (Fig. 4c).

Significant outlier SNPs found by multiple methods were then inspected, either by identifying the exact SNP by more than one approach or by identifying other SNPs in the same genomic region. There were 128 outlier SNPs identified by both DAPC and pcadapt (i.e. 33% of the DAPC outliers and 2.9% of pcadapt outliers). Six genomic scaffolds each contain at least 5 of these 128 common outliers (Table 1; Supplementary Additional File 2). This includes 83 SNPs on scaffold NW_026622875 between 19.86 and 19.97 Mbp (Table 1). Scaffold NW_026622863 has multiple regions with common outliers, including near 47.88 and 56.63 Mbp. There were 21 SNPs identified by both pcadapt and GEMMA, with 11 of these being on NW_025522863 between 47.75 and 47.82 Mbp (Table 1). Although no SNPs were identified by all 3 methods, there were consistently identified regions shared by all 3 approaches, including most notably the region around 47.8 Mbp of NW_026622863 (Table 1). A full list of SNPs associated as identified by each method, and shared loci between methods, is present in Supplementary Additional File 2.

**Table 1.** Genomic regions identified by multiple outlier detection methods with at least 5 loci on the same scaffold that were identified by at least 2 methods.

Scaffold	Methods	Shared loci	Positions Mbp (count)	Genes in region
NW_026622786	DAPC & pcadapt	11	18.56 to 18.59 (4); 25.96 to 25.98 (7)	DPYD (25.20 to 25.99)
NW_026622797	DAPC & pcadapt	5	14.79 to 14.93 (4); 25.49 (1)	CCDC170 (14.77 to 14.88), ARMT1 (14.90 to 14.91), RMND1 (14.91 to 14.95), U4 (14.93)
NW_026622863	DAPC & pcadapt	5	47.87 to 47.88 (2); 56.63 (3)	MARK4 (47.86 to 47.89), CKM (47.89 to 47.90); OZF-like (56.62 to 56.62), FRP2 (56.63 to 56.65)
NW_026622863	GEMMA & pcadapt	11	47.75 to 47.82 (11)	PPP1R37 (47.72 to 47.77), NKPD1 (47.77 to 47.77), TRAPPC6A (47.78 to 47.79), BLOC1S3 (47.79), EXOC3L2 (47.83 to 47.85)
NW_026622875	DAPC & pcadapt	83	19.86 to 19.97 (83)	SLC9A9 (19.61 to 20.16)
NW_026622952	DAPC & pcadapt	7	28.50 to 28.51 (6); 28.60 (1)	WDR17
NW_026622997	DAPC & pcadapt	5	7.64 to 7.65 (5)	SERPINB1 (7.64 to 7.65)

Regions are shown with Mbp positions of the region and the number of shared outlier SNPs in the region in parenthesis (i.e. count). Genes within 10 kbp of the identified regions are shown, and those discussed in-text are underlined and acronyms given below the table. All significant outliers, shared outliers between methods, and gene acronyms are given in Supplementary Additional File 2.

CKM, creatine kinase, M-type; OZF-like, zinc finger protein OZF-like; SLC9A9, solute carrier family 9 member A9.

**Fig. 5.**
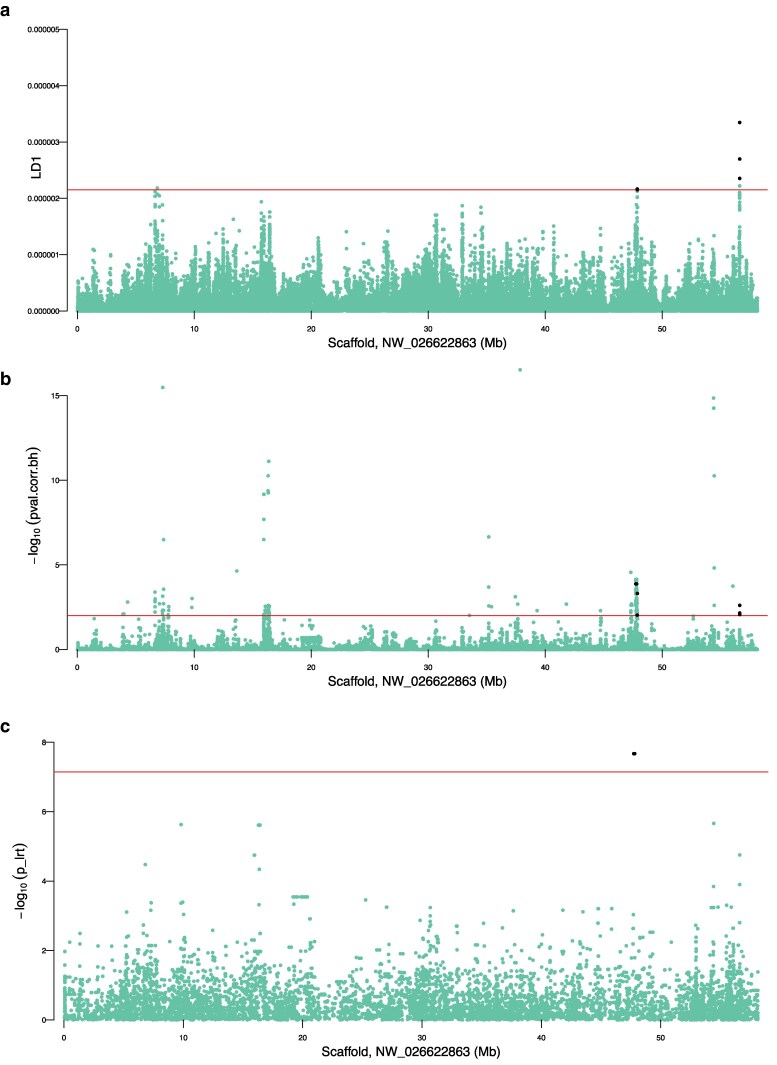
Top outlier region on genomic scaffold NW_026622863, identified as having clusters of outlier loci as detected by all 3 methods near 47 to 48 mbp, shown for a) DAPC (*n* = 2); b) pcadapt (PC2 only; *n* = 96); and C) GEMMA (*n* = 11). Significance cutoffs are described in the full chromosome Manhattan plot figure caption. Black dots are highlighted SNPs that have been identified by multiple methods.

**Fig. 6.**
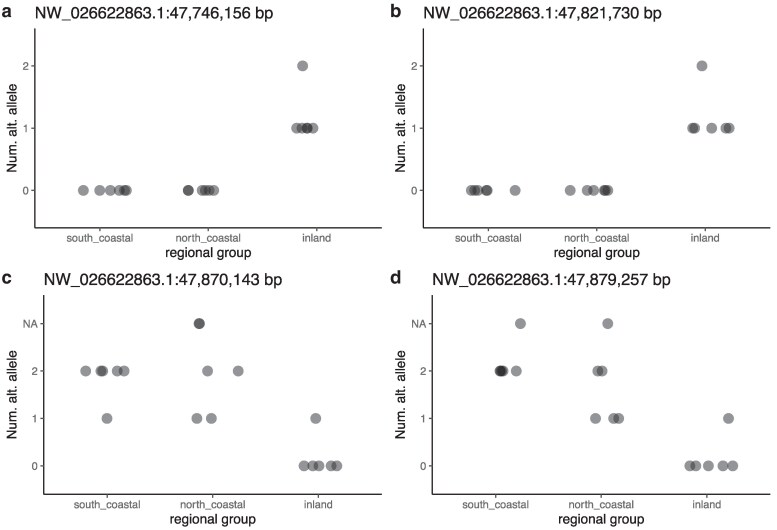
Selected significant outlier SNPs consistently identified by multiple methods within the region of interest on scaffold NW_026622863 shown as the number of alternate alleles for the genotype of each individual from the 3 identified regional groupings. NA values indicate missing genotype data for a sample.

The consistently identified outlier genomic regions were inspected for gene content using the gene annotation for UrsArc2.0 (accessed 2025 June 11). The region of interest identified by all 3 methods, NW_025522863 between 47,746,156 and 47,879,257 bp is a 133,101 bp region that contains 2, 96, and 11 significant SNP outliers for DAPC, pcadapt, and GEMMA, respectively (Fig. 5). This region contains 7 predicted genes (see Table 1). Most notably, at 47,888,920 to 47,897,638 (9.7 kb downstream) is the annotated gene *creatine kinase, M-type* (CKM; Table 1). Several top significant SNPs in this region that were found by multiple outlier detection methods were plotted for allelic dosage (Fig. 6), including pcadapt and GEMMA outliers, including the most significant pcadapt outliers for PC2, a SNP at 47.746 Mbp, and one at 47.822 Mbp. These 2 SNPs show similar genotypic patterns where all coastal individuals are homozygous for the reference allele and most inland individuals are heterozygous (with one being homozygous alternate). Shared DAPC and pcadapt significant outliers were also identified in this region, including a SNP at 47.870 Mbp and one at 47.880 Mbp (Fig. 5; Fig. 6).

The other region on scaffold NW_025522863 identified by both pcadapt and DAPC at 56.63 Mbp is near a zinc finger gene (*zinc finger protein OZF-like*), and multiple other zinc finger genes upstream (i.e. 11 unique predicted zinc finger genes from 56.40 to 56.62 Mbp). The region with 83 shared SNPs by both pcadapt and DAPC at 19.86 to 19.97 Mbp of NW_026622875 is near a predicted gene annotated as *solute carrier family 9 member A9* (Table 1).

## Discussion

Genomic investigations of SNP variants putatively under selection have identified locally adapted populations across small geographic areas (Richardson et al. 2014), in wide-ranging highly mobile species (Schweizer et al. 2016b), and in the presence of gene flow (Tigano and Friesen 2016). Our findings of substructure within the BC sampling range and the presence of putative outlier loci across the coastal mountain range (CMR) indicate the potential for local adaptation in grizzly bears in BC. Additionally, the dataset presented here provides a valuable new genomic resource for grizzly bears that fills a sampling gap through western Canada's Central Coast in the recently published global analysis of whole-genome resequencing of brown bears (de Jong et al. 2023).

### Population genomic trends in BC grizzly bears

The present data showed clustering of grizzlies by the geographic region of sampling (i.e. coastal north, coastal south, and interior), with some individuals having unclear or mixed genetic backgrounds. This clustering was observed after removing individuals with elevated estimated genetic relatedness, which was important given the potential impact of closely related individuals on the tools applied here (i.e. PCA, ADMIXTURE; Patterson et al. 2006; Anderson and Dunham 2008; Elhaik 2022; Yao and Ochoa 2023). The greatest genetic differentiation (i.e. substructuring) in the dataset was based on latitude, not on coastal or interior delineations. Pairs of grizzlies were found with elevated relatedness, and these pairs were sampled in geographically proximal locations, but in some cases, many years apart. Furthermore, increased genetic similarity in proximal geographic areas was independent of year sampled, suggesting that these genetic neighborhoods have persisted over time, even though there are some individuals present with putatively mixed genetic backgrounds among the clusters, and therefore some gene flow could be expected.

Although there was substantial genome-wide *F*_ST_ observed between the 3 genetic clusters (most notably between the southern and northern clusters), the ADMIXTURE analysis found *K* = 1 as the best model to explain the data. This observation suggests that all the clusters originate from the same overall genetic ancestry but may have been impacted by genetic drift relatively recently, resulting in allele frequency changes in the individual clusters, with some gene flow likely (based on the presence of individuals with mixed ancestry fractions with a *K* = 3 ADMIXTURE model). Isolation-by-distance (IBD) can challenge the analytic approaches used here; ADMIXTURE assumes random mating, but IBD can violate this assumption (Lawson et al. 2018), which in some cases can lead to an overestimation of *K* (Frantz et al. 2009). However, in the present study, spatial separation does not appear to have overestimated *K*. It was valuable here to use these multiple different approaches to understand the genetic trends in the study (Lawson et al. 2018).

The genetic differentiation among geographic regions was contrasted by the presence of individuals with mixed ancestry fractions (when using *K* = 3) that were not clearly assigned to any one of the 3 identified clusters. The area around Bella Coola had several sampled individuals with putatively mixed genetic backgrounds that were therefore not included in the outlier identification. The Bella Coola Valley bisects the CMR and therefore may provide a corridor for access to the coastal area. Similarly, an individual with a putatively mixed genetic background was observed on the edge of the designated coastal region near the Kitimat and Kemano valleys connecting the interior to the coastal region. Grizzlies were also observed further into the interior region with mixed *K* = 3 ancestry fractions. The movement of interior grizzly bears through the Bella Coola valley to access salmon has long been known by the Nuxalk Nation (Jason Moody, *pers. comm*.). If valleys traversing the CMR are used as corridors, this provides opportunities for connectivity maintenance for management. These results would benefit from additional samples to improve our understanding of the extent of connectivity and movement during reproductive seasons. Although the present study was limited to the tissues that were available, a more continuous and expanded sampling strategy could improve our understanding of connectivity among these regions, including the potential presence of any significant barriers. Henson et al. (2021) also identified 3 separate groupings (described as STRUCTURE populations) in BC, and these also followed a trend of separating by geography, with some overlap and the presence of putative migrant individuals in the different areas.

Bears are expected to move at different times of year, for example to access salmon in the fall, and for mating in the spring. This natural movement process would provide an important link between coastal and interior habitats. Interestingly, Bella Coola and Kitimat/Kemano also align with 2 of the most important and widely used eulachon *Thaleichthys pacificus* grease trails used and maintained to connect Indigenous communities in trade and other processes over millennia (Harrington 1953). Such convergence in use between species emphasizes how humans and bears can be similarly shaped by landscapes (Henson et al. 2021), and the long-term importance of these valleys for bears and people to access coastal resources.

### Local adaptation and putative outlier loci across the coastal-to-interior ecotone

Although latitude was the most significant explanatory factor for genetic variation, by contrasting interior and coastal regions, outlier loci were detected that provide candidate genomic regions potentially associated with phenotypic differences observed between the coastal and interior regions. Genomic regions with clusters of SNPs identified by multiple approaches were of particular interest, most notably the region from 47.75 to 47.88 Mbp of scaffold NW_026622863.1. This region is 9.7 kbp upstream from the single copy gene *creatine-kinase, m-type* (CKM). This gene is expressed predominantly in muscle and heart tissues of grizzlies of both sexes, as observed based on exon expression profiles in NCBI (PRJNA413091; Jansen et al. 2019). CKM is the muscle type of creatine kinases, and is involved in energy homeostasis, catalyzing the reversible transfer of phosphate from ATP to creatine to produce phosphocreatine (Abnous and Storey 2007), a temporary energy storage in muscle (Whiteman et al. 2017). During food shortages, polar bears reduce activity and use stored energy (e.g. in spring following winter food deprivation); alongside the reduced muscle protein concentration and increased water content that occurs during atrophy, reduced expression of *ckm* mRNA is also observed (Whiteman et al. 2017). In the ground squirrel *Spermophilus richardsonii*, CK activity and protein levels are reduced during hibernation, and *ckm* mRNA expression is reduced by 70% (Abnous and Storey 2007). The physiological role of CKM in energy metabolism associated with intermittent food availability and stores makes this gene an interesting candidate given its proximity to the most consistently identified outlier region between coastal and interior grizzly bears here. This genomic region, and other candidate regions, including the second peak further downstream on the same scaffold that is within a region replete with zinc finger protein-encoding genes (often involved in transcription regulation; Cassandri et al. 2017), merit further investigation in future studies in terms of their potential roles in the differential phenotypes observed between coastal and interior grizzly bears.

Known phenotypic differences exist between coastal and interior BC grizzly bears, and these different regions across the coastal–interior ecotone have significant ecosystem differences to which the residents would be exposed. Key phenotypic and environmental differences have been documented between larger, salmon- and intertidal-foraging coastal bears and smaller, interior bears in terms of morphology (Rausch 1963; Kurtén 1973; Paetkau et al. 1998), resource use (Adams et al. 2017), and potential pathogen pressure (Catalano et al. 2015; Robbins et al. 2018). In coastal bears, adaptations for enhanced growth may have arisen in response to their greater access to, consumption of, and size-mediated competition over salmon (Gende and Quinn 2004; Robbins et al. 2007; Service et al. 2019). In contrast, growth inhibition in interior bears would be advantageous for regulating body mass, given local intermittent access to high-protein resources (Felicetti et al. 2003). The differential pathogen pressures presented by either primarily cervid- or salmon-associated meat resources in interior and coastal areas, respectively, could also result in immune-related adaptations in each area (Catalano et al. 2015; Robbins et al. 2018). The outlier loci identified in the present study may be related to these phenotypic and ecotypic differences, including resource niche differentiation of coastal and interior grizzly bears. However, they are likely only part of a complex suite of polygenic and epigenetic differences that interact with diet-induced patterns of phenotypic plasticity.

Increased understanding of the underlying environmental factors that drive local adaptation can help to identify loci associated with local adaptation (Booker 2024). Improved characterization and analytic use of the drivers of the main selective forces on grizzlies across the ecotone may therefore improve our ability to detect loci associated with this selection. Without an exact characterization of the environment that each grizzly experienced for extended periods of time, the present study relied upon sampling location to contrast the different subpopulations (with an attempt to exclude putative migrants). If an environmental variable suspected of being a driver of selection across the ecotone was known, and the per-grizzly value of this variable was obtained and usable in the association analysis, this could improve resolution of the genomic associations to the ecotone. However, it is not clear whether this exact specification of a continuous variable per individual would be possible for grizzlies, considering their wide-ranging habitats, and is not possible with the current dataset, and therefore we relied upon general geographic groupings that were classified as either coastal or interior. Understanding selection can also be improved by considering dispersal patterns alongside environmental variation (Booker 2024). Importantly, when selective pressures and population structure are co-autocorrelated over geographic areas (as could be expected in grizzlies across the CMR ecotone), local adaptation can be strong (depending on gene flow and strength of selection), but it may also be more difficult to characterize (Booker 2024). The present study gives initial insights into this question across the BC CMR ecotone in grizzly bears.

Increased sample sizes from each region of the study would improve resolution and reduce the potential for false positive outlier detection. In addition to the relatively low sample size, another potential shortcoming of our approach is the grouping of southern and northern coastal samples together to compare with the interior samples (that are more genetically similar to the northern coastal cluster than the southern coastal). This approach assumes that the 2 coastal regions, although they have the greatest differentiation in the dataset, will have had parallel adaptations or consistent genotypic variation across the ecotone. There is also a possibility of confounding latitudinal variation with ecotone-related variation, although inspections of individual loci for top outliers show consistent genotypes in both southern and northern coastal areas contrasted with the interior. Removing the southern coastal samples and only analyzing the coastal-to-interior contrast at a similar latitude would reduce the sample size by a third, and therefore be expected to significantly reduce detection power. Additionally, the north-to-south variation was mainly captured by a separate axis of variation in the pcadapt analysis. In any case, the findings of regions putatively linked to the alternate sides of the coastal-to-interior ecotone are valuable for future studies but should also be considered as initial evidence for involvement and not definitive. Further evaluating the associations of these regions with segregating phenotypic variation across the coastal-to-interior ecotone will be important in future work.

### Management implications

Our results indicating population substructure and potential local adaptation have implications that can be considered for management applications. For example, these results indicate gene flow among provincially designated GBPUs, as well as the potential for locally adapted regional groups. Individuals from the genetically continuous interior group span multiple current GBPUs (i.e. Tweedsmuir and Bulkley GBPUs; Fig. 1), which emphasizes the need to maintain connectivity among these management units. Furthermore, the GBPU system may not adequately describe, integrate, and protect corridors bisecting the CMR to connect coastal and interior groups. Although the restricted geographic sampling in the present study limits inference regarding the spatial designation of coastal GBPUs, evidence for coastal latitudinal genetic differentiation was observed. Although it was not formally evaluated here given the focus on the coast-to-interior ecotone, the present data could also be investigated for outliers between the 2 coastal regions, albeit with a reduced sample size to the present analysis.

Interior grizzlies have adapted to more extreme environmental conditions found in continental climates and have regulations on body size presumably related to intermittent resources (Robbins et al. 2007). Coastal individuals may lack such adaptations to maintain body size, which could pose a risk under a future defined by ever decreasing populations of salmon (Price et al. 2008). With increased variation in environmental conditions expected, adaptations for these fluctuating conditions may not be present in the coastal group (Felicetti et al. 2003). The immunological capacities may also differ between regions; for example, interior individuals may lack immunity to pathogens present in the coastal environment presented by intertidal and marine protein resources (Catalano et al. 2015; Robbins et al. 2018). The possibility of these vulnerabilities and the demonstrated susceptibility elsewhere of locally adapted populations to environmental stressors (Valladares et al. 2014; Anderson and Wadgymar 2020) highlights the value of future research investigating local adaptation in these regions, as well as the frequency and extent of gene flow. Our findings also support cautious management practices designed to preserve gene flow between coastal and interior groups and protect salmon and coastal habitats as resources linked to the evolutionary history and future productivity of potentially uniquely adapted coastal grizzly bears.

## Conclusions

Here, we used whole-genome resequencing to improve our understanding of the genetic differences across the coastal-to-interior ecotone in BC, and in doing so identified 3 distinct subpopulation clusters (i.e. north coastal, south coastal, and interior). By inspecting grizzlies identified as predominantly belonging to each of the 3 subpopulations, we identified segregating genetic variants and associated genomic regions and candidate genes between the coastal and interior regions and signatures of potential local adaptation. With continued environmental or resource changes in each region, local adaptation will be important to consider in terms of resiliency of grizzly bears from different geographic regions. These results suggest that it will be important for management to consider both the connectivity corridors between regions, but also the potential for locally adapted and unique subpopulations depending on the geographic region.

## Supplementary Material

jkaf237_Supplementary_Data

## Data Availability

Raw sequence data have been uploaded to NCBI's short read archive (SRA) under BioProject PRJNA1204358 within accessions SAMN46039649-SAMN46039679. Datashare agreements with the Province of British Columbia restrict the sharing of precise locations where samples were obtained. VCF files containing sample multilocus genotypes are available through the G3 FigShare portal (https://doi.org/10.25387/g3.30090427). The following code repositories support this project: Manuscript code repository: https://github.com/bensutherland/ms_grizzly_popgen. Population genetics analysis: https://github.com/bensutherland/simple_pop_stats. Additional bioinformatics functions: https://github.com/enormandeau/Scripts. Supplemental material available at G3 online.
