# Supplementary material for: Grizzly bear population genomics across a coastal–interior ecotone in British Columbia, Canada
Source: G3 (Bethesda). 2025 Oct 7;15(12):jkaf237. doi: 10.1093/g3journal/jkaf237 (PMC12693532; doi:10.1093/g3journal/jkaf237)
Supplement: jkaf237_Supplementary_Data [file jkaf237_supplementary_data.zip › Supplemental_Material_G3-2025-406083.docx]

**Additional Files**

Additional File S1. Sampling metadata, read numbers, alignment rates, and missing data per individual.

Additional File S2. Significant or top outlier loci as identified by supervised DAPC, pcadapt, and GEMMA, including shared outliers between methods, and gene of interest acronyms.

Supplemental Results include Figures S1-S9 and Table S1-S2.
