# Supplementary material for: Grizzly bear population genomics across a coastal–interior ecotone in British Columbia, Canada
Source: G3 (Bethesda). 2025 Oct 7;15(12):jkaf237. doi: 10.1093/g3journal/jkaf237 (PMC12693532; doi:10.1093/g3journal/jkaf237)
Supplement: jkaf237_Supplementary_Data [file jkaf237_supplementary_data.zip › Supplemental_Results_G3-2025-406083.docx]

**Supplemental Information for:**

**Grizzly bear population genomics across a coastal-interior ecotone in British Columbia, Canada**

Lauren H. Henson, Kris A. Christensen, Ben J. G. Sutherland, Hollie A. Johnson, Bridgett vonHoldt, Astrid Vik Stronen, Paul Paquet, Jason Moody, Ben F. Koop, Chris T. Darimont

**Table of Contents:**

| **Figure S1. Per individual missing data** | **Page 2** |
| --- | --- |
| **Figure S2. Mitochondrial DNA phylogenetic tree** | **Page 3** |
| **Figure S3. PCA considering (A) sex and year; (B) missing data** | **Page 4** |
| **Figure S4. DAPC variance and BIC** | **Page 5** |
| **Figure S5. Unsupervised DAPC density plot and assignplot** | **Page 6** |
| **Figure S6. ADMIXTURE CV error (*K* = 1 - 6)** | **Page 7** |
| **Figure S7. ADMIXTURE barplot** | **Page 7** |
| **Figure S8. Supervised coastal vs. interior DAPC density plot** | **Page 8** |
| **Figure S9. pcadapt scoreplot, screeplot, and qq plot** | **Page 9** |
| **Table S1. Pairs of grizzlies with elevated relatedness** | **Page 10** |
| **Table S2. *F*_ST_ table** | **Page 10** |


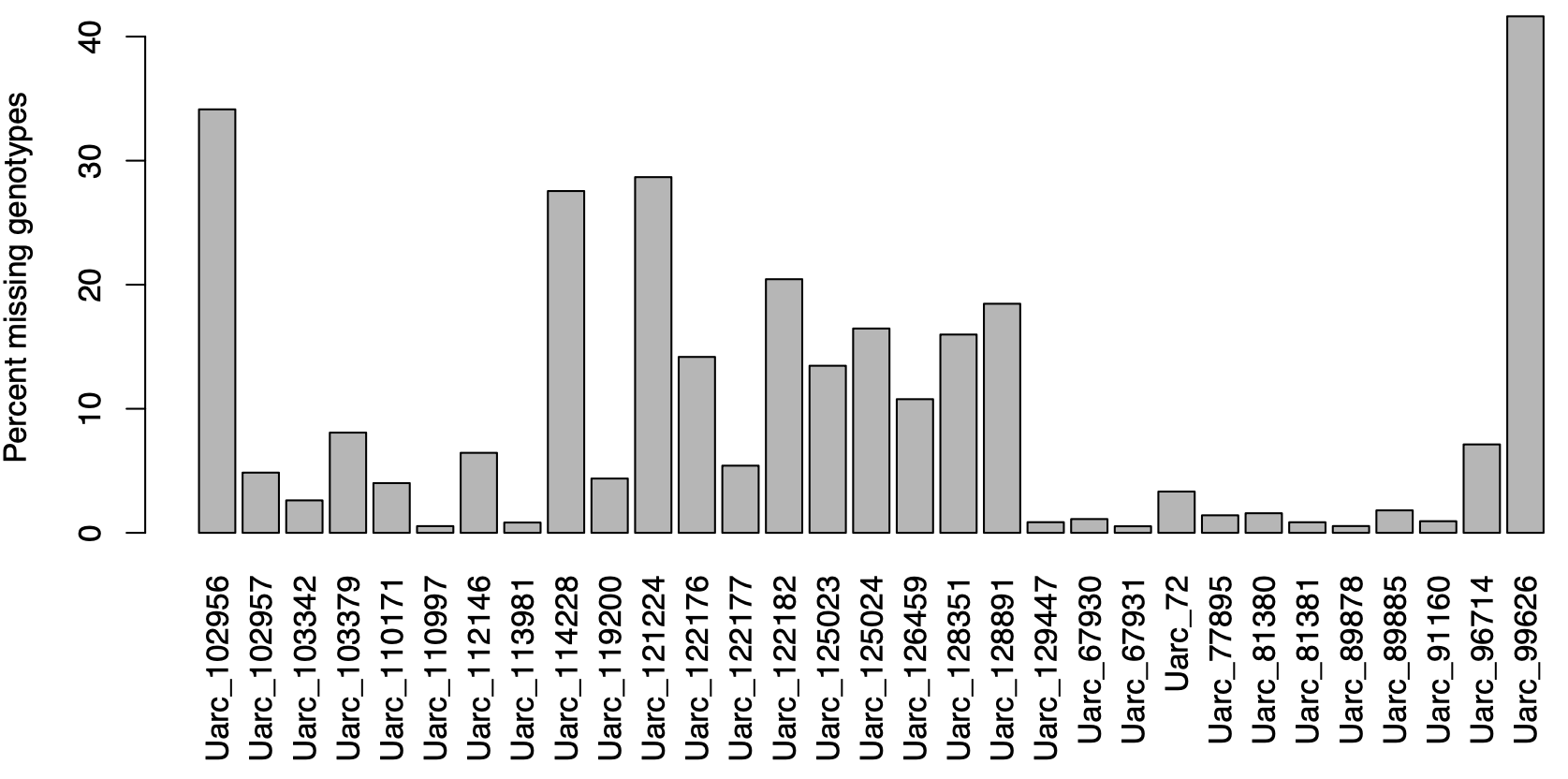


**Figure S1.** Per-individual percentages of missing data in the LD-filtered SNP dataset, following all filters.


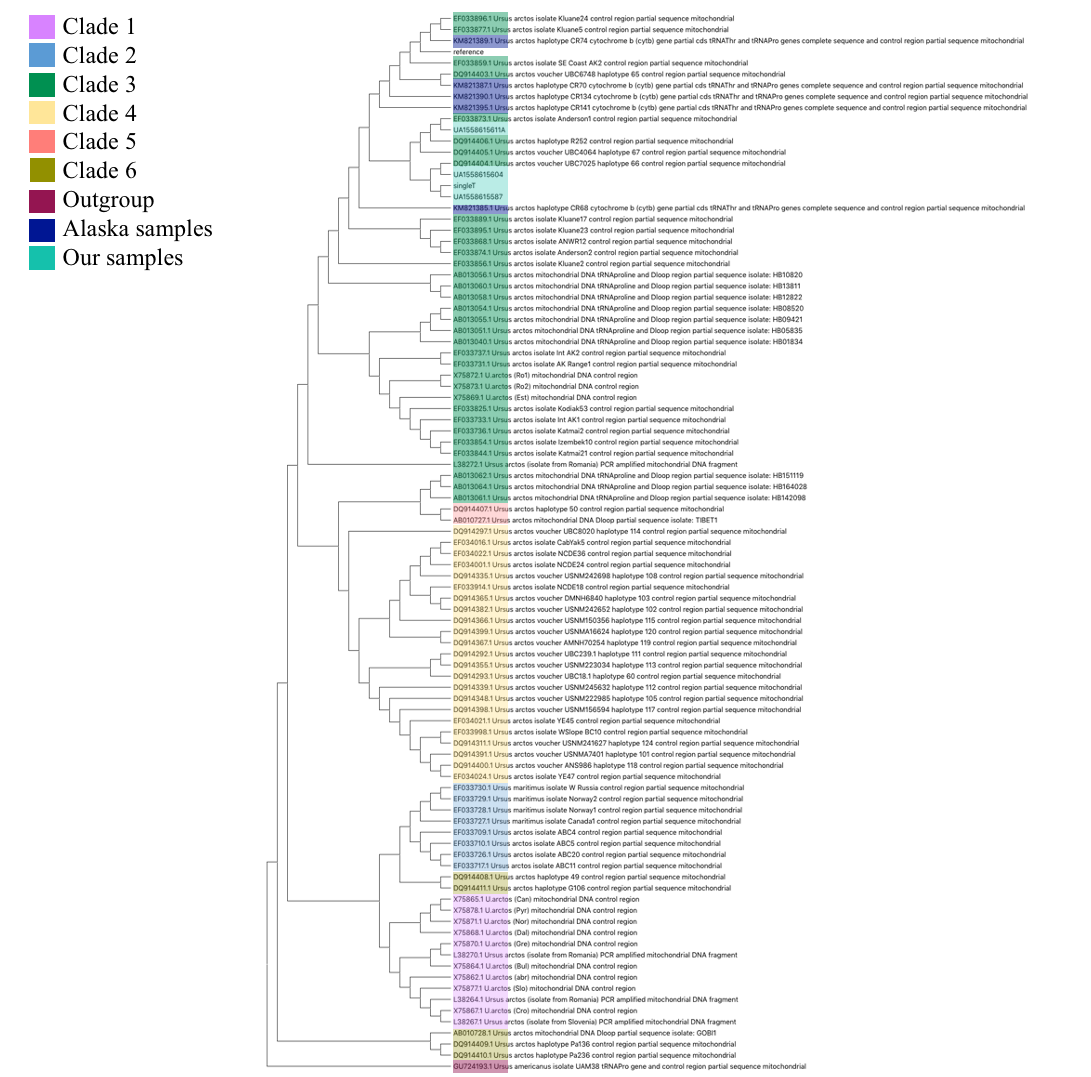


**Figure S2.** Highest log likelihood MEGA X generated Maximum Likelihood tree with *Ursus americanus* outgroup. Individuals representing each haplotype are distinguished by NCBI identifier where applicable. Described clades reflect clades identified in Miller et al. (2006).

**(A)**


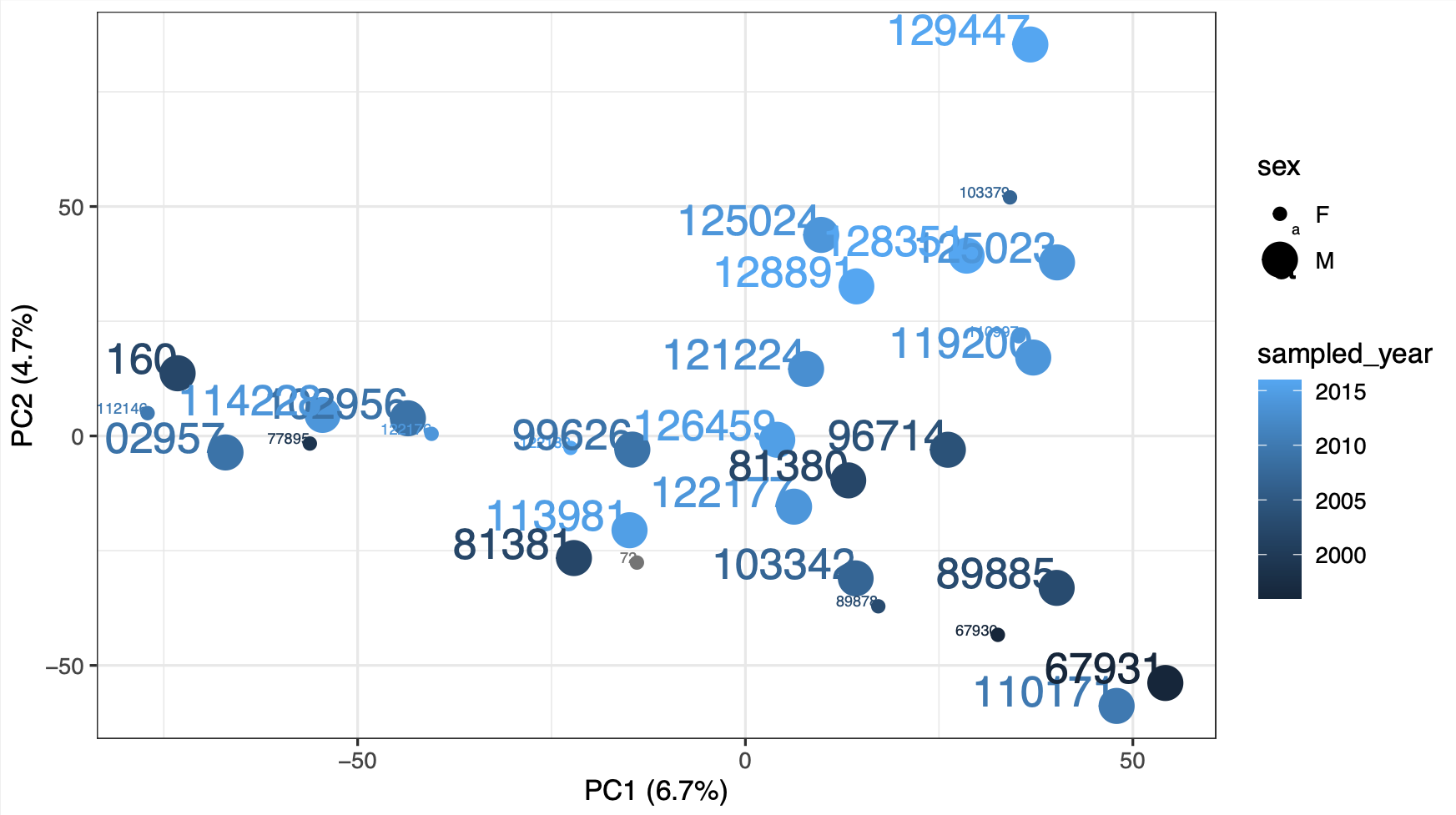


**(B)**


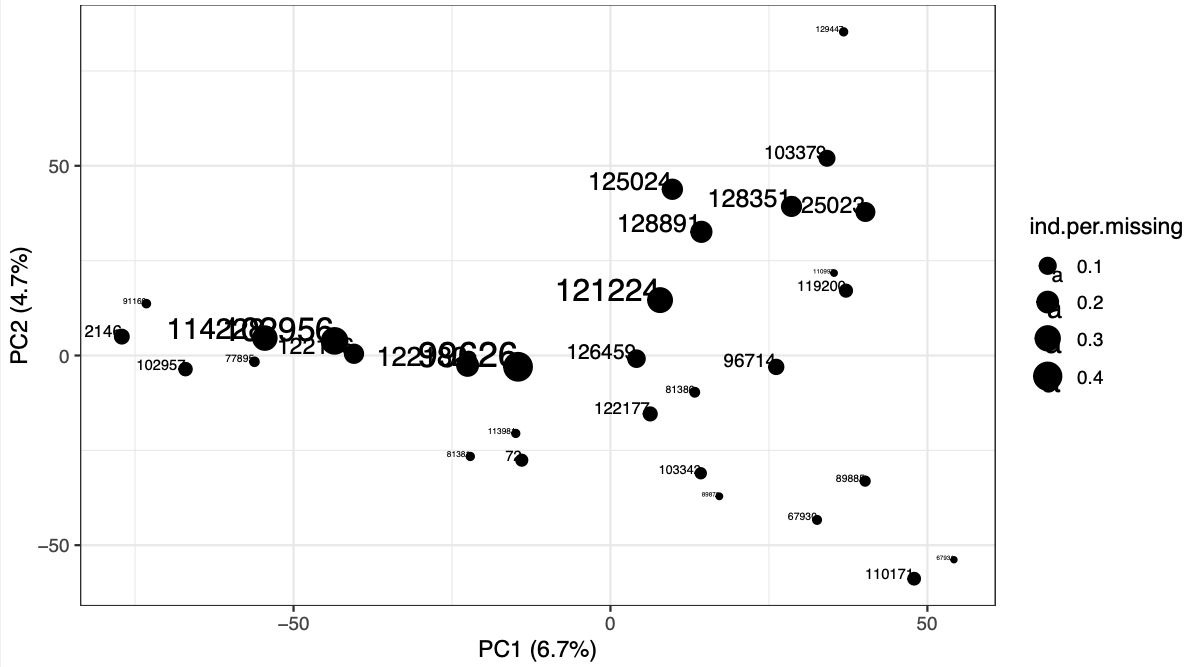


**Figure S3**. Samples clustered by principal components analysis (PCA) based on LD-filtered genotypes showing (A) sex and sampling year; and (B) proportion of missing data per individual following all filters.

**(A) (B)**


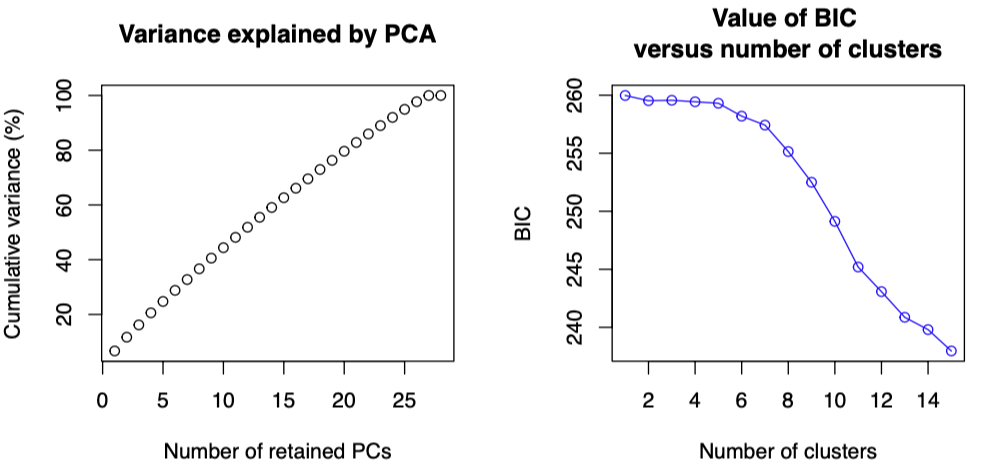


**Figure S4.** (A) Variance explained with number of retained principal components in unsupervised DAPC and (B) value of BIC with increasing numbers of clusters.

**(A)**

**
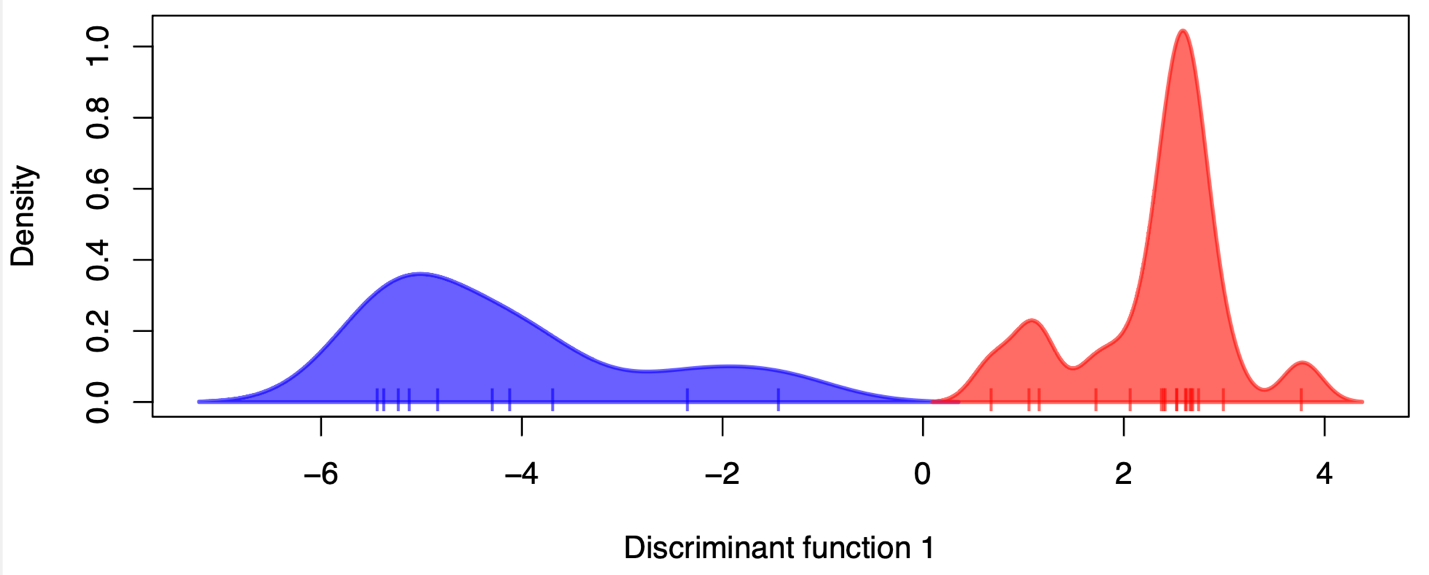
**

**(B)**


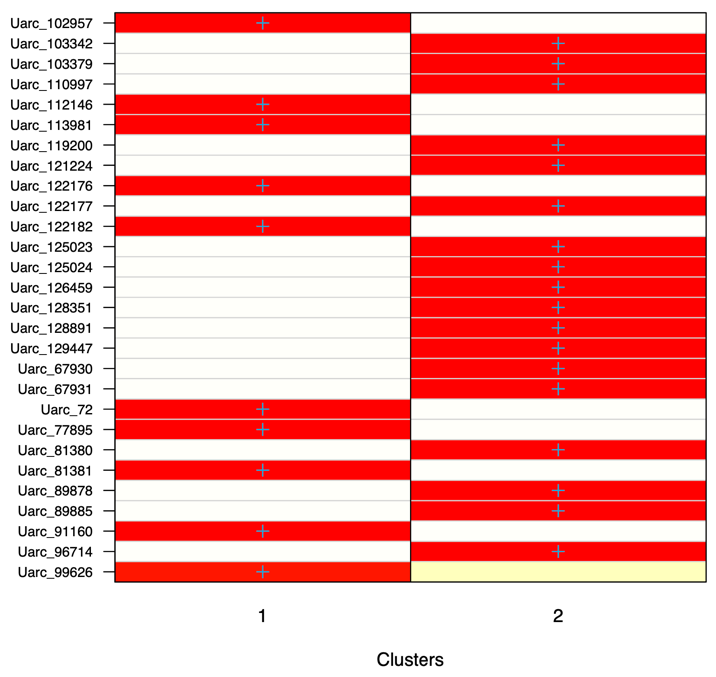


**Figure S5.** Unsupervised DAPC with *K* = 2 (A) density plot; and (B) sample assignment plot.


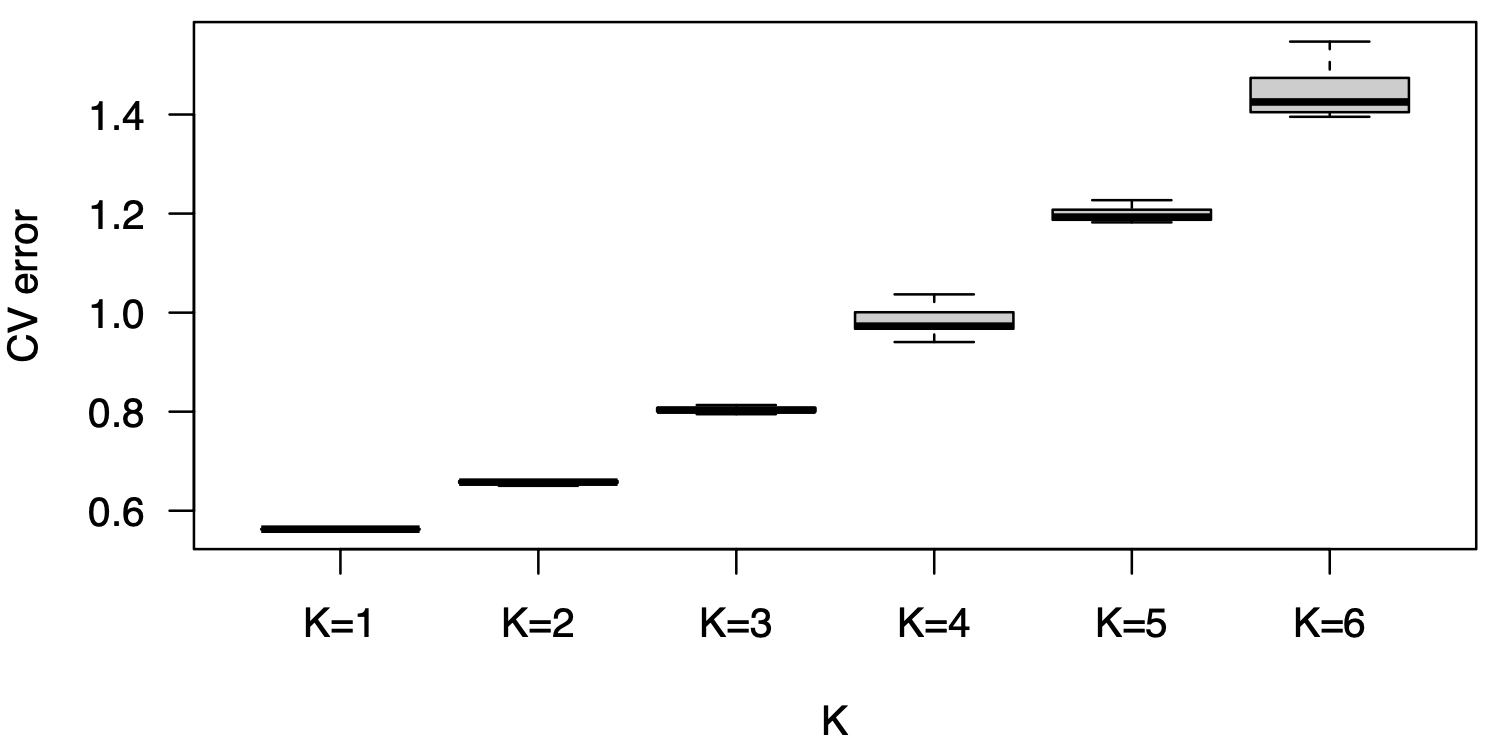


**Figure S6.** ADMIXTURE CV error for *K* = 1 through 6 to identify the value of *K* with the lowest CV error.


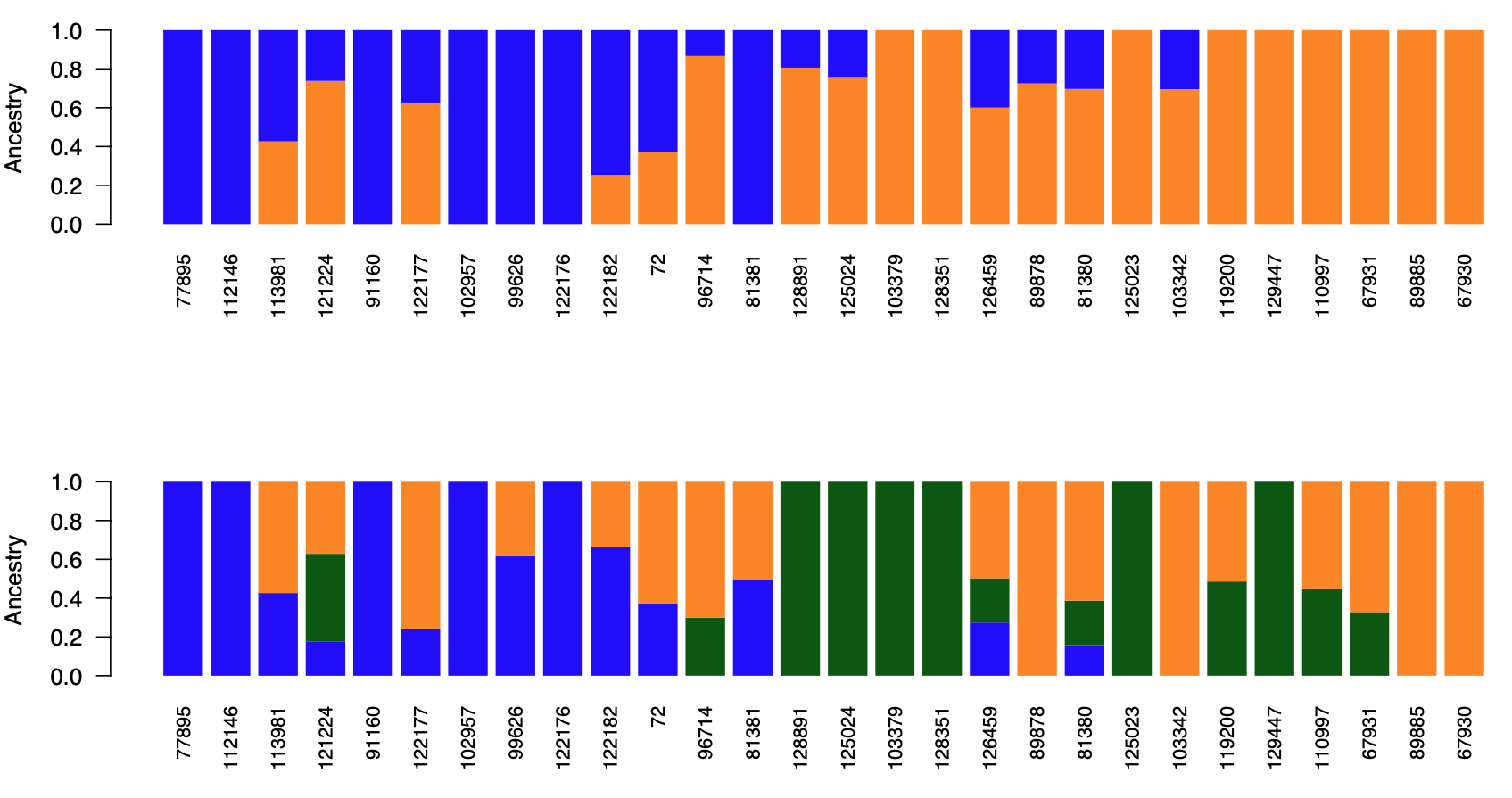


**Figure S7.** ADMIXTURE barplot with *K* = 2 (upper) and *K* = 3 (lower) showing proportions of ancestry fractions per sample.


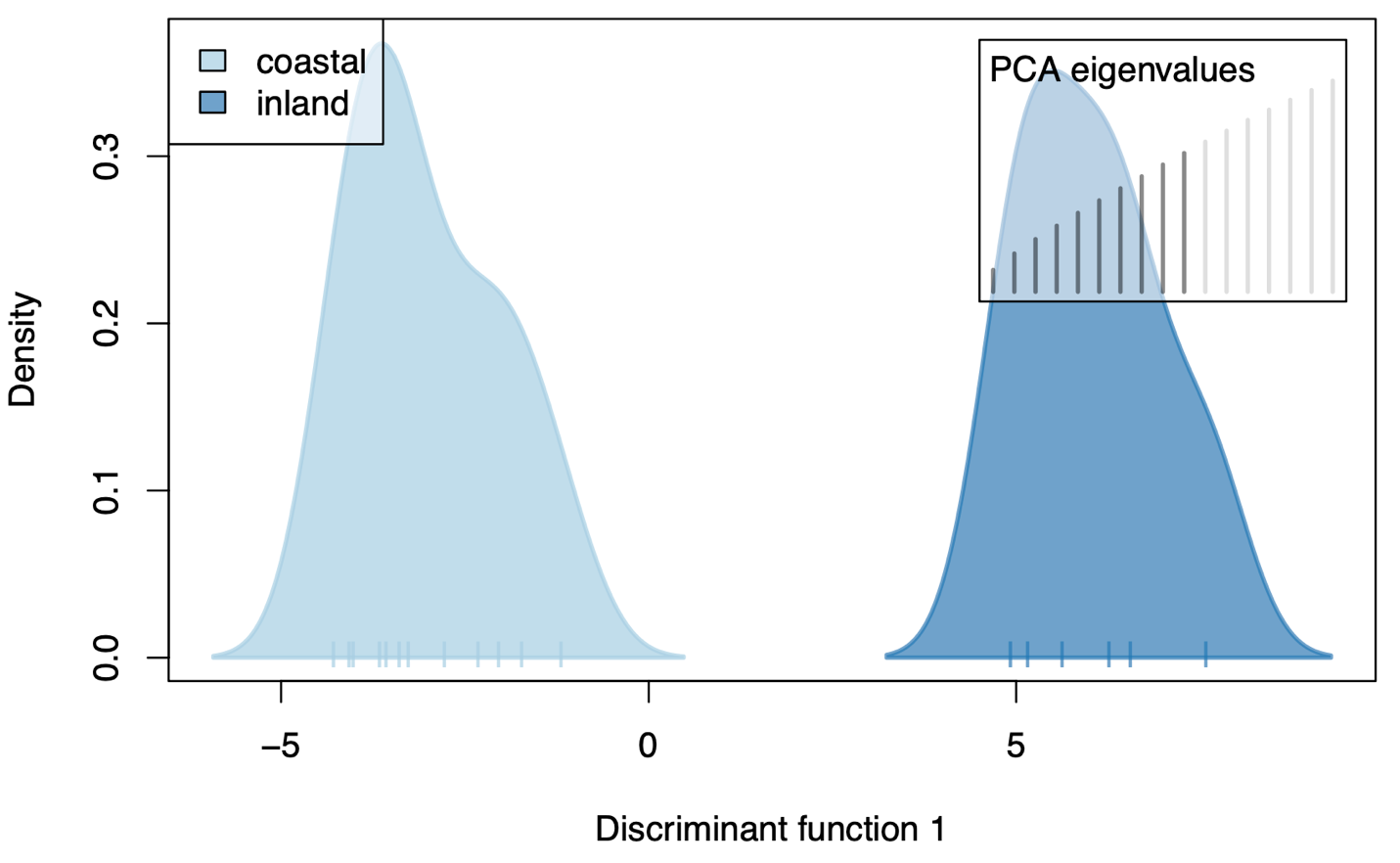
**Figure S8.** Supervised DAPC (coastal vs. inland) density plot.

**(A)**


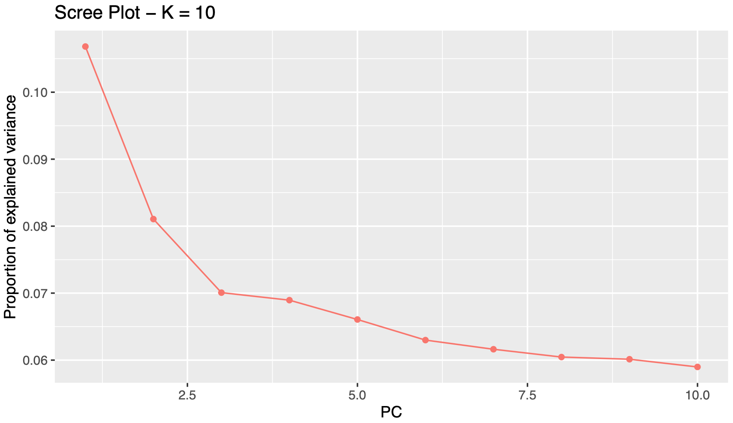


**(B)**


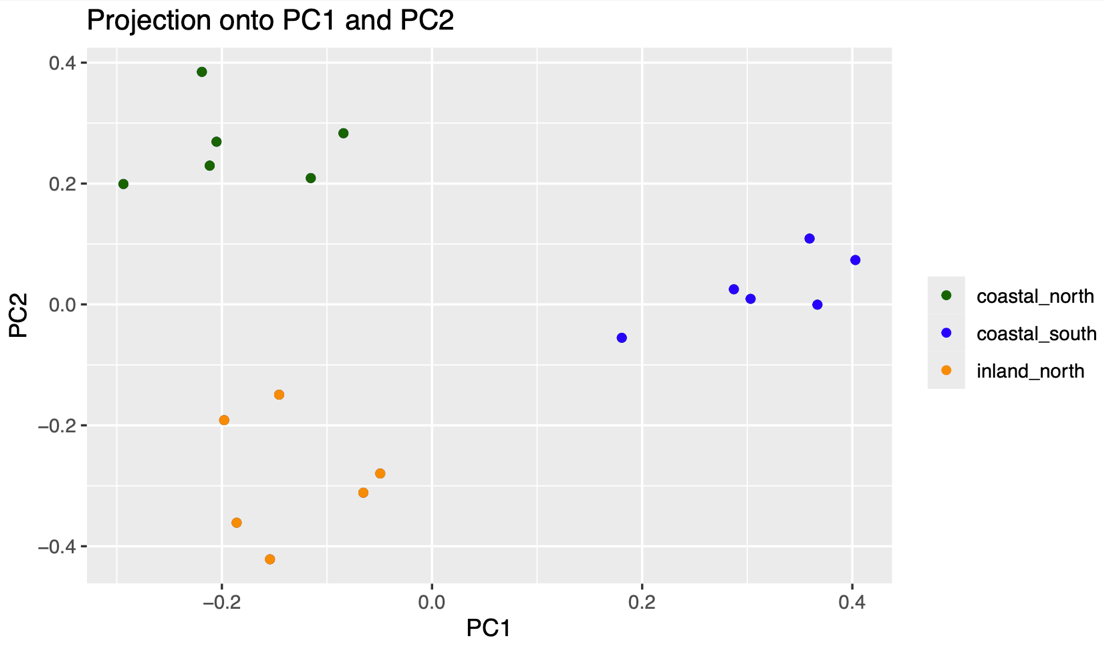


**(C)**

­
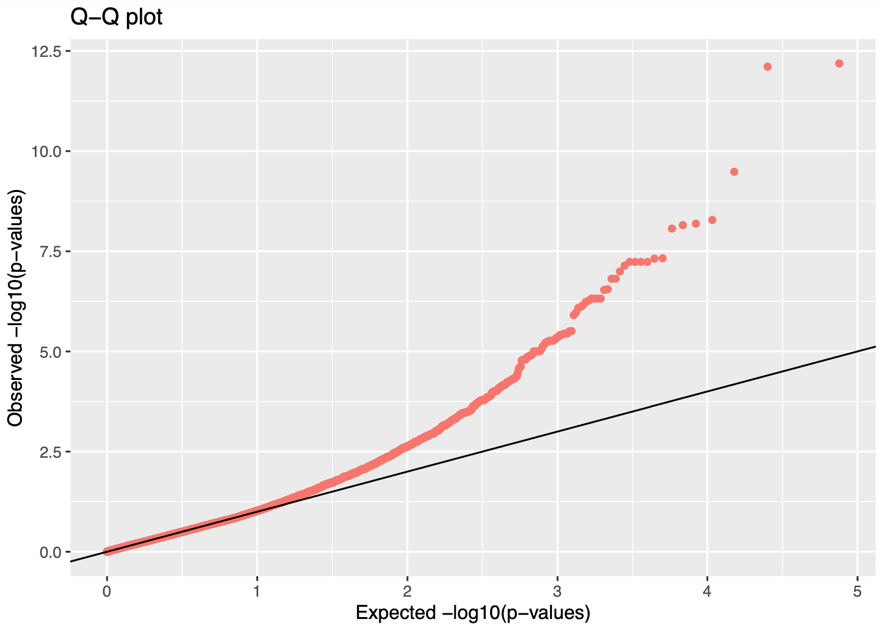


**Figure S9.** pcadapt (A) Scree plot; (B) projection of samples on PC1 and PC2; and (C) Q-Q plot.

**Table S1.** Pairs of samples with highest calculated relatedness values (Ritland statistic > 0.15). The individual with the asterisk was removed from the dataset to avoid impacts of putative close-kin. Distance between calculated by ground length in Google Earth based on sample jittered GPS coordinates.

| **Indiv. 1** | **Indiv. 1,**  **sex and year** | **Indiv. 2** | **Indiv. 2,**  **sex and year** | **Distance between (km)** | **Ritland statistic** |
| --- | --- | --- | --- | --- | --- |
| 110171* | M, 2010 | 67931 | M, 1996 | 61.5 | 0.2471 |
| 112146 | F, 2010 | 114228* | M, 2014 | 11.6 | 0.2304 |
| 102956* | M, 2009 | 114228* | M, 2014 | 23.1 | 0.1705 |
| 102956* | M, 2009 | 112146 | F, 2010 | 22.1 | 0.1529 |
| 114228* | M, 2014 | 91160 | M, 2002 | 27.4 | 0.1512 |

**Table S2.** Genetic differentiation (mean *F*_ST_) between clusters using only individuals that were conclusively assigned to clusters (n = 6 per cluster).

|  | **Coastal, north** | **Coastal, south** | **Interior** |
| --- | --- | --- | --- |
| **Coastal, north** | - |  |  |
| **Coastal, south** | 0.0360 | - |  |
| **Interior** | 0.0149 | 0.0304 | - |
